# Supplementary material for: Diverse prehistoric cattle husbandry strategies in the forests of Central Europe
Source: Nat Ecol Evol. 2024 Oct 29;9(1):87–98. doi: 10.1038/s41559-024-02553-y (PMC11726460; doi:10.1038/s41559-024-02553-y)
Supplement: Supplementary file 1 — Reporting Summary [file 41559_2024_2553_MOESM1_ESM.pdf]

nature portfolio

Corresponding author(s): Rosalind E. GIllis

Last updated by author(s): May 8, 2024

Reporting Summary

Nature Portfolio wishes to improve the reproducibility of the work that we publish. This form provides structure for consistency and transparency in reporting. For further information on Nature Portfolio policies, see our Editorial Policies and the Editorial Policy Checklist.

Statistics

For all statistical analyses, confirm that the following items are present in the figure legend, table legend, main text, or Methods section.

n/a	Confirmed	
☐	☒	The exact sample size (*n*) for each experimental group/condition, given as a discrete number and unit of measurement
☐	☒	A statement on whether measurements were taken from distinct samples or whether the same sample was measured repeatedly
☐	☒	The statistical test(s) used AND whether they are one- or two-sided<br>*Only common tests should be described solely by name; describe more complex techniques in the Methods section.*
☐	☒	A description of all covariates tested
☒	☐	A description of any assumptions or corrections, such as tests of normality and adjustment for multiple comparisons
☐	☒	A full description of the statistical parameters including central tendency (e.g. means) or other basic estimates (e.g. regression coefficient) AND variation (e.g. standard deviation) or associated estimates of uncertainty (e.g. confidence intervals)
☐	☒	For null hypothesis testing, the test statistic (e.g. *F*, *t*, *r*) with confidence intervals, effect sizes, degrees of freedom and *P* value noted<br>*Give P values as exact values whenever suitable.*
☒	☐	For Bayesian analysis, information on the choice of priors and Markov chain Monte Carlo settings
☒	☐	For hierarchical and complex designs, identification of the appropriate level for tests and full reporting of outcomes
☒	☐	Estimates of effect sizes (e.g. Cohen's *d*, Pearson's *r*), indicating how they were calculated

Our web collection on statistics for biologists contains articles on many of the points above.

Software and code

Policy information about availability of computer code

Data collection	Where relevant we have described how the data was collected.
Data analysis	Yes, full description of platforms and programs used to preform the analysis

For manuscripts utilizing custom algorithms or software that are central to the research but not yet described in published literature, software must be made available to editors and reviewers. We strongly encourage code deposition in a community repository (e.g. GitHub). See the Nature Portfolio guidelines for submitting code & software for further information.

Data

Policy information about availability of data

All manuscripts must include a data availability statement. This statement should provide the following information, where applicable:

- Accession codes, unique identifiers, or web links for publicly available datasets
- A description of any restrictions on data availability
- For clinical datasets or third party data, please ensure that the statement adheres to our policy

The data is avaiable as Supplementary tables

Human research participants

Policy information about studies involving human research participants and Sex and Gender in Research.

Reporting on sex and gender	N/A
Population characteristics	N/A
Recruitment	N/A
Ethics oversight	N/A

Note that full information on the approval of the study protocol must also be provided in the manuscript.

Field-specific reporting

Please select the one below that is the best fit for your research. If you are not sure, read the appropriate sections before making your selection.

☐ Life sciences ☐ Behavioural & social sciences ☒ Ecological, evolutionary & environmental sciences

For a reference copy of the document with all sections, see nature.com/documents/nr-reporting-summary-flat.pdf

Ecological, evolutionary & environmental sciences study design

All studies must disclose on these points even when the disclosure is negative.

Study description	The study used stable isotopic measurements of carbon, oxygen and nitrogen from ancient cattle bioapatite, dentine and collagen as well as ruminant dairy fats collected from prehistoric pottery. The relationship between climate and environment was explored using paleoenvironmental proxies.
Research sample	The research sample consisted of stable isotopic measurements of carbon, oxygen and nitrogen from ancient cattle (8000yrs old) bioapatite, dentine and collagen as well as ruminant dairy fats collected from prehistoric pottery (c.8000yrs old).
Sampling strategy	Samples were selected for good preservation of skeletal features. Pottery samples focused on non restored rim sherds.
Data collection	The data was collected by Melanie Roffet-Salque, Iain Kendall, Marco Zanon, Veronika Brychova, Arkadiusz Marciniak, Emmanuelle Casanova and Rosalind Gillis, during the laboratory and data analysis.
Timing and spatial scale	No repetition was made of stable isotopic measurements. These measurements are representable of a temporal period of between 5900BC and 5400BC cal.
Data exclusions	N/A
Reproducibility	The experiment design and methodology is well described and established.
Randomization	N/A
Blinding	N/A

Did the study involve field work? ☒ Yes ☐ No

Field work, collection and transport

Field conditions	Archaeological material was collected during exacavations that have taken place over the last 30 years. The material was then collected by Rosalind Gillis and Melanie Roffet-Salque from various depots and museums across central Europe.
Location	Central Europe
Access & import/export	All of the material has been exported with full permission of local authorities and excavators, most of whom are co-authors on the paper. All studied materials are currently being repatriated where possible.
Disturbance	N/A

Reporting for specific materials, systems and methods

We require information from authors about some types of materials, experimental systems and methods used in many studies. Here, indicate whether each material, system or method listed is relevant to your study. If you are not sure if a list item applies to your research, read the appropriate section before selecting a response.

Materials & experimental systems

n/a	Involved in the study	
☒	☐	Antibodies
☒	☐	Eukaryotic cell lines
☐	☒	Palaeontology and archaeology
☐	☒	Animals and other organisms
☒	☐	Clinical data
☒	☐	Dual use research of concern

Methods

n/a	Involved in the study	
☒	☐	ChIP-seq
☒	☐	Flow cytometry
☒	☐	MRI-based neuroimaging

Palaeontology and Archaeology

Specimen provenance	All of the material has been exported with full permission of local authorities and excavators, most of whom are co-authors on the paper. All studied materials are currently being repatriated where possible. Given the large number of sites, it is not possible to provide full documentation and is available on request.
Specimen deposition	All studied materials are currently being repatriated where possible. Cattle teeth are well preserved and can be used for future analysis. All stable isotopic results are available with this publication.
Dating methods	Radiocarbon dates were provided by the excavators and are summarized in Supplementary table 1.

☒ Tick this box to confirm that the raw and calibrated dates are available in the paper or in Supplementary Information.

Ethics oversight	N/A

Note that full information on the approval of the study protocol must also be provided in the manuscript.

Animals and other research organisms

Policy information about studies involving animals; ARRIVE guidelines recommended for reporting animal research, and Sex and Gender in Research

Laboratory animals	N/A
Wild animals	N/A
Reporting on sex	N/A
Field-collected samples	Ancient cattle and deer teeth and bone collected from archaeological sites.
Ethics oversight	N/A

Note that full information on the approval of the study protocol must also be provided in the manuscript.
